# Dynamic Effects Analysis in Fractional Memristor-Based Rulkov Neuron Model

**DOI:** 10.3390/biomimetics9090543

**Published:** 2024-09-08

**Authors:** Mahdieh Ghasemi, Zeinab Malek Raeissi, Ali Foroutannia, Masoud Mohammadian, Farshad Shakeriaski

**Affiliations:** 1 Neural Engineering Laboratory, Department of Biomedical Engineering, University of Neyshabur, Neyshabur 9319774446, Iran; m.ghasemi@neyshabur.ac.ir (M.G.); zeinabmalekraeissi@gmail.com (Z.M.R.); 2 Faculty of Science and Technology, University of Canberra, Canberra, ACT 2617, Australia; ali.foroutannia@canberra.edu.au (A.F.); masoud.mohammadian@canberra.edu.au (M.M.)

**Keywords:** Rulkov map, discrete memristor, discrete fractional order, chaotic systems, synchronization of two coupled neurons

## Abstract

Mathematical models such as Fitzhugh–Nagoma and Hodgkin–Huxley models have been used to understand complex nervous systems. Still, due to their complexity, these models have made it challenging to analyze neural function. The discrete Rulkov model allows the analysis of neural function to facilitate the investigation of neuronal dynamics or others. This paper introduces a fractional memristor Rulkov neuron model and analyzes its dynamic effects, investigating how to improve neuron models by combining discrete memristors and fractional derivatives. These improvements include the more accurate generation of heritable properties compared to full-order models, the treatment of dynamic firing activity at multiple time scales for a single neuron, and the better performance of firing frequency responses in fractional designs compared to integer models. Initially, we combined a Rulkov neuron model with a memristor and evaluated all system parameters using bifurcation diagrams and the 0–1 chaos test. Subsequently, we applied a discrete fractional-order approach to the Rulkov memristor map. We investigated the impact of all parameters and the fractional order on the model and observed that the system exhibited various behaviors, including tonic firing, periodic firing, and chaotic firing. We also found that the more I tend towards the correct order, the more chaotic modes in the range of parameters. Following this, we coupled the proposed model with a similar one and assessed how the fractional order influences synchronization. Our results demonstrated that the fractional order significantly improves synchronization. The results of this research emphasize that the combination of memristor and discrete neurons provides an effective tool for modeling and estimating biophysical effects in neurons and artificial neural networks.

## 1. Introduction

Mathematical models are needed to understand the principles and mechanisms of complex biological neural network systems with extensive neuronal connections [1,2]. In the past, models such as Fitzhugh-Nagoma [3], Hodgkin–Huxley [4], fusion and leaky fire [5], Hindmarsh–Rose [6], and Morris–Licar [7], which were based on ion channels, were expressed by continuous-time differential equations. However, due to their inherent complexity, these models have challenged the analysis of neural function, and the development of straightforward and approximate time-discrete models has solved these challenges by focusing on specific neural functions [8]. One of the models that has attracted the attention of researchers in this regard is Rulkov’s discrete model, which has made it possible to analyze neural function in a manageable and interpretable way [9].

Nearly three decades before Rulkov’s model was proposed, Chua 1971 introduced the memristor as the fourth non-linear circuit component connecting magnetic flux and electric charge [10]. Physical realization of memristor was performed by HP laboratory in 2008 [11]. The widespread presence of memristors in logic circuits [12], electrical synapse simulation [13], flash memories [14], and neural networks [15] comes from the characteristics of memory and its inherent nonlinearity [16,17]. The presence of these parts in non-linear systems also gives rise to multistability and chaos phenomena [18,19]. Memristors with synaptic plasticity are emerging as the basic blocks of neuromorphic systems [20]. An example of this can be seen in the model of Yao and his colleagues for training neural networks by neuromorphic circuits equipped with memristors [21]. Memristor’s distinctive features and capabilities have attracted considerable attention and excitement among researchers and engineers. As a result, the memristor is increasingly considered a critical component for various applications in neuronal modeling.

Discrete fractional-order derivatives are employed in map-based dynamic analysis to capture the influence of memory on processing, as they offer greater accuracy compared to integer-order derivatives [22,23]. These derivatives retain information about past behaviors in memory [24,25]. Previous research has utilized fractional-order derivatives to model biological systems across various neural networks, physics, and biology applications [26,27,28,29,30]. Fractional derivative properties in the biological sense are expressed as heritable properties with more accuracy in comparing full-order models, treating dynamic firing activities at multiple time scales for a single neuron, better performance of firing frequency responses in fractional designs than integer models, enriching functional neural mechanisms [31,32,33].

Using a neuron involved with a memristor when the action potential in the neuron causes an induced electromagnetic current [34] can effectively record and describe the effect of electromagnetic induction [35]. Based on this idea, we presented a discrete memristor with hyperbolic tangent inductance, and then we implemented memory effects on it with the help of discrete fractional-order derivatives. In each step, we examined the dynamic behaviors of the modeled system. Then, we evaluated the effects of synchronization between two neurons as a collective phenomenon between reciprocal units evaluated on the discrete memristor-Rulkov (m-Rulkov) fractional-order model in such a way that the neuroscience evaluation of the synchronization of neural networks represents the origin of the emergence of oscillating rhythms with different cognitive tasks.

The study proceeds as follows: In Section 2, we introduce the Rulkov map, the discrete memristor with flux control feature, and the discrete m-Rulkov, which serves as the foundation of our study. Section 3 evaluates the dynamic effects of the m-Rulkov model parameters. Section 4 focuses on simulating the impact of fractional order and system parameters by formulating the discrete m-Rulkov fractional-order model to explore the system dynamics. Section 5 discusses the synchronization of two Rulkov neurons by the discrete memristor that plays a role in the synapse position. Finally, in Section 6 of this study, we conclude.

## 2. Mathematic Model

### 2.1. Rulkov’s Model

Rulkov’s two-dimensional model was introduced in 2001 by Nikolai F. Rulkov to study neural networks [9]. This iterative map has a simple algebraic structure, a low-dimensional phase space, and less computational burden than continuous-time differential equations. It can imitate various neural activities observed in real neurons, such as silence regime, tonic spiking, burst spiking, and chaos [9,36,37]. This system has two fast and slow dynamics to produce fast excited explosions on top of slow oscillations in the following format [9]:

(1) $$\left\{\begin{array}{c} x\left(n+1\right)=f\left(x\left(n\right),y\left(n\right)\right)\\ y\left(n+1\right)=y\left(n\right)-µ\left(x\left(n\right)-\sigma +1\right) \end{array}\right.,$$

where $x\left(n\right)$ (representative of fast dynamics) is the excitatory variable of neuron membrane voltage, $y\left(n\right)$ (representative of slow dynamics) represents the ion concentration, and as the recovery variable, μ is the control parameter, and σ is the externally imposed effect. The discontinuous and non-linear function $f\left(x\left(n\right),\;y\left(n\right)\right)$ with the control parameter, α is expressed as follows [9,36]:

(2) $$f\left(x\left(n\right),\;y\left(n\right)\right)=\left\{\begin{array}{cc} \frac{\alpha }{1-x(n)}+y\left(n\right) & x(n)\leq 0\\ \alpha +y(n) & 0<x(n)<\alpha +y(n)\\ -1 & x(n)\geq \alpha +y(n) \end{array}\right..$$

**Figure 1:**
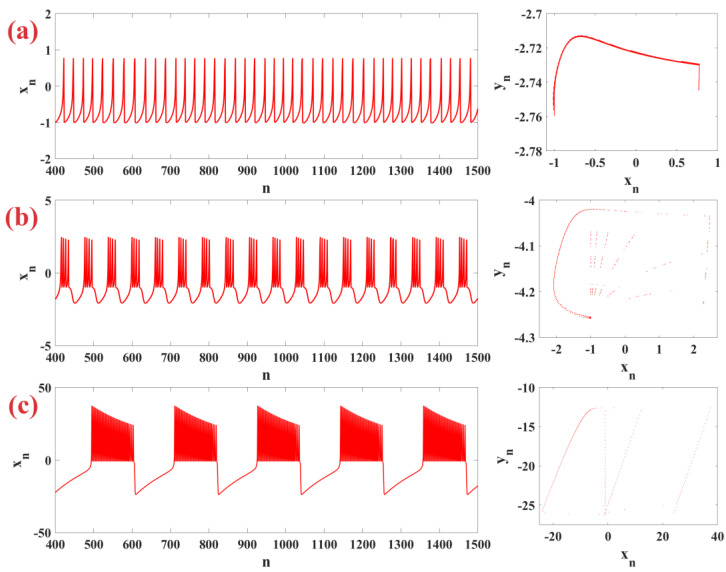
Time series and phase plane representation of neuronal firing patterns produced by Rulkov neuron model with a constant value of μ=0.01 in (**a**) tonic firing mode for α=3.5 and σ=0.3, (**b**) periodic firing mode for α=6.5 and σ=0, and (**c**) chaotic firing mode for α=50 and σ=0.05.

By setting μ=0.01 and changing parameters α and σ, the time series and phase plane of different neural firing patterns are shown by the Rulkov map in Figure 1. In part (a), the tonic firing for the neuron with α=3.5 and σ=0.3 as a continuous and regular firing pattern, part (b) the periodic firing for a neuron with α=6.5 and σ=0, and part (c) the chaotic periodic firing for a neuron with α=50 and σ=0.05 are shown.

### 2.2. Discrete Memristor

Voltage, current, charge, and magnetic flux are the four primary variables in electrical circuits, and there is always a relationship between the two components. The relationship between electric charge and magnetic flux as the fourth two-terminal circuit element after resistance, capacitor, and inductor was introduced by Leon O. Chua in 1971 as a memristor [10]. An ideal flux-controlled memristor with hyperbolic tangent inductance can be modeled as follows [38]:

(3) $$\left\{\begin{array}{c} i=W\left(\varphi \right)v=\tanh\left(\varphi \right)v\\ \frac{d\varphi }{d\tau }=v \end{array}\right.,$$

where v, i, φ, and ꚍ are the variables of voltage, current, magnetic flux, and time, respectively, where $W\left(\varphi \right)=\tanh\left(\varphi \right)$ is the memristor inductance. Memconductance describes the memory behavior of a device or component, which refers to the device’s ability to control conductivity (ability to pass electrical current) based on the history of applied voltage or current [10]. By changing the flux along the length of the memristor, the inductance of the memristor moves between these two states, and this movement causes the current and voltage to change throughout the memristor, which means the intrinsic and unique nonlinearity of the memristor. Due to memory-dependent conductivity, a memristor that acts like a memory resistor, unlike regular resistors, its voltage–current curve does not follow a linear relationship, and its voltage–current relationship creates hysteresis curves due to the stated switching. This characteristic of memristors has caused them to become an efficient element in modeling non-linear circuits and chaotic systems [17]. Now, the memristor we want to create must simulate the electromagnetic induction of the neuron’s action potential. Since Rulkov’s discrete model is used in this article to simulate the neuron, the memristor by which the effects of electromagnetic induction are studied must also be discretized. To obtain the discrete memristor, we discretize the continuous memristor model (Equation (3)) as follows:

(4) $$\left\{\begin{array}{c} i\left(n\right)=W\left(\varphi \left(n\right)\right)\;v\left(n\right)=\tanh\left(\varphi \left(n\right)\right)v\left(n\right)\\ \varphi \left(n+1\right)=\varphi \left(n\right)+\varepsilon v\left(n\right) \end{array}\right.$$

where $\varphi \left(n+1\right)$ is the value of φ(t) in step n+1. Additionally, the parameter ε is used as a time scale factor to create the induced driving force in a limited transition period to investigate the effect of electromagnetic induction in the neuron. The parameter ε depends on the nature of the environment and is a material coefficient used to create more chaos and explosive shots by setting it to 1 and 0.05, respectively [36,38].

### 2.3. Discrete Memristor-Rulkov Model

In this section, by integrating the Rulkov neuron model (Equation (1)) with the discrete memristor (Equation (4)), we present the discrete m-Rulkov model to study the effects of electromagnetic induction. If the electromagnetic induction is expressed by the expression $k\;tanh\left(\varphi \left(n\right)\right)\;x\left(n\right)$, the m-Rulkov map with electromagnetic induction effects will be displayed in the following order:

(5) $$\left\{\begin{array}{c} x\left(n+1\right)=f\left(x\left(n\right),y\left(n\right)\right)+k\;tanh\left(\varphi \left(n\right)\right)\;x\left(n\right)\\ y\left(n+1\right)=y\left(n\right)-\mu \left(x\left(n\right)-\sigma +1\right)\\ \varphi \left(n+1\right)=\varphi \left(n\right)+\varepsilon x\left(n\right) \end{array}\right.,$$

where the action potential $x\left(n\right)$ induces a current with the induction power k. Exposing it to an external magnetic field disrupts the ion pumping, and it can manipulate the magnetic flux and induced current. As a result, the mentioned model works more efficiently in detecting state changes and evaluating the effect of physical fields on neural firing behaviors caused by variable magnetic fields.

## 3. Dynamics Analysis in Discrete m-Rulkov Model

In the following, the effect of electromagnetic induction on the system dynamics is studied using the bifurcation diagram and the zero-one chaos test for the parameters of the equations. The zero-one test is a simple approach with a binary nature that distinguishes regular and chaotic dynamics. This technique uses time series data to examine the convergence and divergence of near paths in a chaotic system. The zero-one test can be calculated with two regression and correlation methods. This study focuses on the correlation method because it shows a better result than the regression method compared to the weak chaos. To calculate the zero-one chaos test in a sequence $N_{k}$, $k=0,\;1,\ldots ,\bar{N}–1$, the variables $p_{c}$ and $q_{c}$ are calculated as the following expressions:

(6) $$q_{c}(n)=\sum_{j=1}^{n}N_{j}\sin\left[\left(j+1\right)c\right],n=1,2,3,\ldots ,\bar{N}–1$$

where $c\;\epsilon \;\left(0,\;\pi \right)$, and the behavior of the variables $p_{c}$ and $q_{c}$ in terms of diffusion and non-diffusion can be determined by using the concept of mean square displacement $M_{c}\left(n\right)$, $n=0,\;1,\ldots ,n_{cut}$ with the correct recommended value $n_{cut}\approx \frac{\left(\bar{N}-1\right)}{10}$ evaluated as follows:

(7) $$M_{c}\left(n\right)=\frac{1}{N}\sum_{j=1}^{N}{\left[p_{c}\left(j+n\right)-p_{c}\left(j\right)\right]}^{2}+{\left[q_{c}\left(j+n\right)-q_{c}\left(j\right)\right]}^{2}.$$

The mean square displacement remains bounded for systems with regular dynamics as time progresses. This means that the system’s displacement from its initial position does not grow indefinitely but instead reaches a limit or stabilizes over time. In contrast, in systems with chaotic dynamics, the mean square of the displacement shows a linear relationship with the time scale, indicating that the system’s displacement from its initial position increases continuously and proportionally with time. This linear relationship shows the exponential growth of the system’s displacement, indicating chaotic behavior. By forming the vectors $\Delta =[M_{c}\left(0\right),\;M_{c}\left(1\right),\;M_{c}\left(2\right),\;\ldots ,\;M_{c}\left(n_{cut}\right)]$ and $S=1,\;2,\;3,\;\ldots ,\;n_{cut}$ and calculating the covariance and variance in the usual way, the correlation coefficient is defined as follows:

(8) $$K_{c}=corr\left(S,\Delta \right)=\frac{cov\left(S,\Delta \right)}{\sqrt{var\left(S\right)var(\Delta )}}\in [-1,\;1],$$

where $K_{c}\approx 0$ reveals regular behavior and $K_{c}\approx 1$ reveals chaotic behavior. The $K_{c}$ value obtained gives us insight into the dynamic nature of the system, through which we can distinguish between orderly and chaotic dynamics in a system [39].

### 3.1. Equilibrium Points and Stability Analysis

Checking the stability of a discrete map is obtained by calculating the matrix Jacobian eigenvalues of the map’s equilibrium points [40,41,42]. According to the definition of the balance point, the balance point is where the output of the map does not change in the next iteration and is the same as its current output, assuming $\left(x^{*},y^{*},\varphi^{*}\right)$ as a fixed point and replacing $\left(x\left(n\right),y\left(n\right),\varphi \left(n\right)\right)$ and $\left(x\left(n+1\right),y\left(n+1\right),\varphi \left(n+1\right)\right)$ in Equation (5).

(9) $$x^{*}=f\left(x^{*},y^{*}\right)+k\tanh\left(\varphi^{*}\right)\;x^{*}$$

(10) $$y^{*}=y^{*}-\mu \left(x^{*}-\sigma +1\right)$$

(11) $$\varphi^{*}=\varphi^{*}+\varepsilon x^{*}$$

(12) $$f(x^{*},y^{*})=\left\{\begin{array}{cc} \frac{\alpha }{1-x^{*}}+y^{*} & x^{*}\leq 0\\ \alpha +y^{*} & 0<x^{*}<\alpha +y^{*}\\ -1 & x^{*}\geq \alpha +y^{*} \end{array}\right.$$

Based on Equation (11), we conclude that $x^{*}=0$, and also based on Equation (10), $x^{*}=\sigma -1$. Two equilibrium points are determined for $x^{*}$, which by putting in Equation (9) based on the conditions of Equation (12), we have as follows.

- If $x^{*}\leq 0$

For the equilibrium point we have $x^{*}=0$:

(13) $$x^{*}=0\to \alpha +y^{*}=0\to y^{*}=-\alpha$$

As a result, the equilibrium point is $\left(0,-\alpha ,m\right)$, where m is an arbitrary fixed value, as a result, there are infinite fixed points. For the equilibrium point we have $x^{*}=\sigma -1$:

(14) $$x^{*}=\sigma -1\to \sigma -1=\frac{\alpha }{2-\sigma }+y^{*}+k\tanh\left(\varphi^{*}\right)\left(\sigma -1\right)\to y^{*}=\frac{\alpha }{2-\sigma }+k\tanh\left(\varphi^{*}\right)\left(\sigma -1\right)-\sigma +1$$

As a result, the equilibrium point is $\left(0,\frac{\alpha }{2-\sigma }+k\tanh\left(m\right)\left(\sigma -1\right)-\sigma +1,m\right)$, and as a result there are infinite fixed points.

- If $0<x^{*}<\alpha +y^{*}$

Considering that $x^{*}=0$ is not in the desired interval, the calculation of the values does not make sense, so we only have $x^{*}=\sigma -1$ for the equilibrium point:

(15) $$x^{*}=\sigma -1\to \sigma -1=\alpha +y^{*}+k\tanh\left(\varphi^{*}\right)\left(\sigma -1\right)\to y^{*}=\alpha -\sigma +1+k\tanh\left(\varphi^{*}\right)\left(\sigma -1\right)$$

As a result, the equilibrium point is $\left(0,\alpha -\sigma +1+k\tanh\left(m\right)\left(\sigma -1\right),m\right)$, so there are infinite fixed points.

- If $x^{*}\geq \alpha +y^{*}$

Considering that $x^{*}=0$ is not in the desired interval, the calculation of the values does not make sense, so we only have $x^{*}=\sigma -1$ for the equilibrium point:

(16) $$x^{*}=\sigma -1\to \sigma -1=-1+k\tanh\left(\varphi^{*}\right)\left(\sigma -1\right)\to \sigma =k\tanh\left(\varphi^{*}\right)\left(\sigma -1\right)\to \tanh\left(\varphi^{*}\right)=\frac{\sigma }{k\left(\sigma -1\right)}\to \varphi^{*}=\tanh^{-1}\left(\frac{\sigma }{k\left(\sigma -1\right)}\right)$$

As a result, the equilibrium point is $\left(0,m,\tanh^{-1}\left(\frac{\sigma }{k\left(\sigma -1\right)}\right)\right)$, and as a result there are infinite fixed points. To check the stability, the Jacobian matrix of the m-Rulkov map is used at the equilibrium point as follows:

- If $x^{*}\leq 0$
  (17) $$Jacobian=\left[\begin{array}{ccc} k\tanh\left(\varphi^{*}\right)+\frac{\alpha }{{\left(x^{*}-1\right)}^{2}} & 1 & -kx^{*}\left(\tanh^{2}\left(\varphi^{*}\right)-1\right)\\ -\mu & 1 & 0\\ \varepsilon & 0 & 1 \end{array}\right]$$
- If $0<x^{*}<\alpha +y^{*}$
  (18) $$Jacobian=\left[\begin{array}{ccc} k\tanh\left(\varphi^{*}\right) & 1 & -kx^{*}\left(\tanh^{2}\left(\varphi^{*}\right)-1\right)\\ -\mu & 1 & 0\\ \varepsilon & 0 & 1 \end{array}\right]$$
- If $x^{*}\geq \alpha +y^{*}$
  (19) $$Jacobian=\left[\begin{array}{ccc} k\tanh\left(\varphi^{*}\right) & 0 & -kx^{*}\left(\tanh^{2}\left(\varphi^{*}\right)-1\right)\\ -\mu & 1 & 0\\ \varepsilon & 0 & 1 \end{array}\right]$$

By calculating the eigenvalues of the Jacobian matrix at fixed points, we can understand whether those points are stable or unstable. When the eigenvalues λ of the fixed point lie on the unit circle, the fixed point is stable. When any eigenvalue of λ is outside the unit circle, the fixed point is unstable. Considering that we have infinite equilibrium points, it is not possible to check all points, and stability and instability are determined based on the stated conditions.

### 3.2. Effects of Inductive Power k

**Figure 2:**
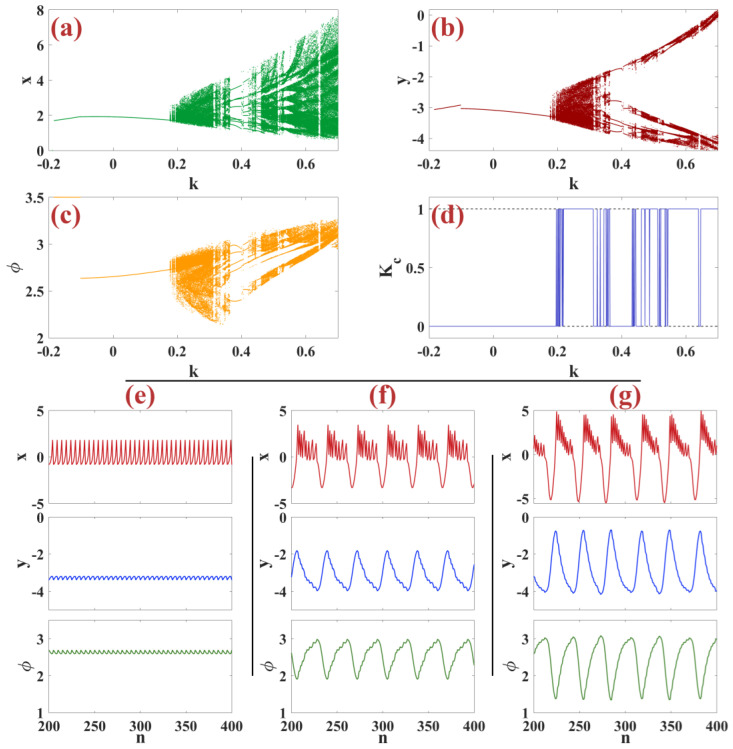
(**a**–**c**) Bifurcation diagrams of x, y, and φ, and a chaotic zero-one test diagram based on the change of induced power *k* for random initial conditions in the interval [−1, 1]; (**d**) chaotic zero-one test diagram in random condition; (**e**–**g**) Neural firing patterns in x, y and φ for tonic spike behavior, periodic firing, chaotic firing.

In this subsection, we examine the dynamic analysis of the induced power parameter for the m-Rulkov neuron map using bifurcation diagrams and the zero-one test. Bifurcation diagram by changing parameter k in the range −0.2 to 0.7 with random initial conditions in the range [−1, 1] for the variable $x\left(n\right)$ in part (a), for the variable $y\left(n\right)$ in part (b), and for the variable $\varphi \left(n\right)$ in part (c) of Figure 2 are shown, and the chaos test 0–1 is shown in part (d) of Figure 2 so that all parameters are set as α=5, μ=0.1, ε=0.05, σ=1. The behavior of the neuron starts with the silence regime. It continues with the progression of k from k=−0.2 until reaching k=0.19, after which it turns into tonic firing at k=0.19 and then enters the area where the firings chaotic and periodic are successively repeated and finally settled in chaos so that the results of the zero-one test diagram follow the behavior of the bifurcation diagram. By adjusting k, the m-Rulkov model can mimic different neural firing patterns such as silence, tonic firing, intermittent, and chaotic firing. Figure 2 shows in parts (e, f, g) the neural firing patterns of x, y, and φ variables for the tonic spike, periodic firing, and chaotic firing patterns.

### 3.3. Effects of Control Parameter α

**Figure 3:**
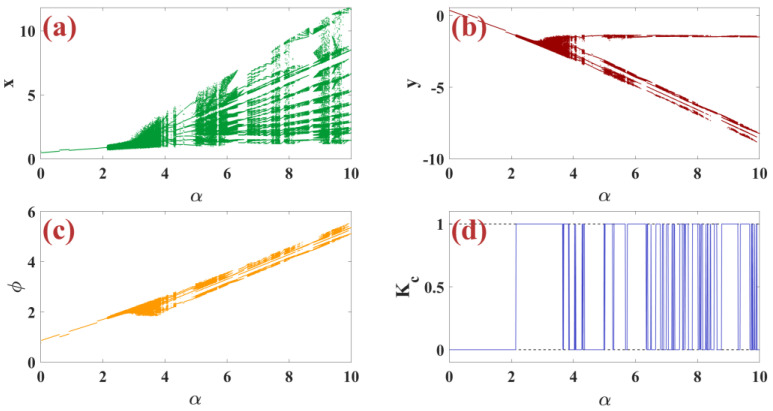
(**a**–**c**) Bifurcation diagrams of x, y, and φ based on the change of the control parameter α with the (**d**) chaotic zero-one test diagram per random condition.

In this subsection, we examine the dynamic analysis of the control parameter α for the m-Rulkov neuron map using bifurcation diagrams and the zero and one chaotic test. Bifurcation diagram by changing the parameter α in the range from 0 to 10 with random initial conditions [−1, 1] for the variable $x\left(n\right)$ in part (a), for the variable $y\left(n\right)$ in part (b), and for the variable $\varphi \left(n\right)$ in part (c) of Figure 3 are shown. Additionally, the test diagram of zero and one chaos is shown in part (d) of Figure 3. So that all parameters are set as μ=0.1, ε=0.05, σ=1, k=0.46. Bifurcation diagrams with silence from α=2.292 are the beginning of tonic firing. With the increase of α, it can be seen that tonic firing and chaotic firing are created successively, eventually leading to chaos. The results of the zero-one test are consistent with the behavior of bifurcation diagrams.

### 3.4. Effects of Control Parameter ε

**Figure 4:**
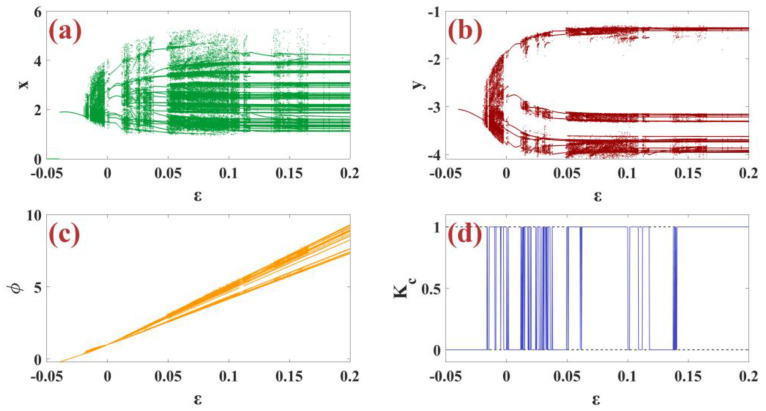
(**a**–**c**) The x, y, and φ bifurcation diagrams are based on changing the control parameter ε with (**d**) chaotic zero-one test diagram per random condition.

In this subsection, we examine the dynamic analysis of the control parameter ε for the m-Rulkov neuron map using bifurcation diagrams and the zero and one test. Bifurcation diagram with changing parameter ε in the range −0.05 to 0.2 with random initial conditions in the range [−1, 1] for the variable $x\left(n\right)$ in part (a), for the variable $y\left(n\right)$ in part (b), and for the variable $\varphi \left(n\right)$ in part (c) of Figure 4 are shown. Additionally, the test diagram of zero and one chaos is shown in part (d) of Figure 4. So that all parameters are set as α=5, μ=0.1, σ=1, k=0.46.

### 3.5. Effects of Externally Imposed Effect σ

**Figure 5:**
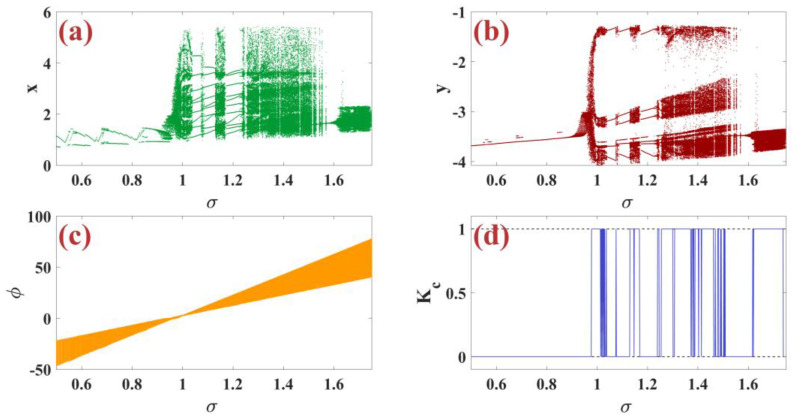
(**a**–**c**) Bifurcation diagrams of x, y, and φ based on the change of the externally imposed effect parameter σ with (**d**) chaotic zero-one test diagram per random condition.

In this subsection, we examine the dynamic analysis of the externally imposed effect parameter σ for the m-Rulkov neuron map using bifurcation diagrams and the zero-one test. Bifurcation diagram by changing the parameter σ in the range of 0.5 to 1.75 with random initial conditions in the range [−1, 1] for the variable x(n) in part (a), for the variable y(n) in part (b), and for the variable φ(n) in part (c) of Figure 5 are shown. Additionally, the test diagram of zero and one turbulence is shown in part (d) of Figure 5. So that all parameters are set as α=5, μ=0.1, ε=0.05, k=0.46.

### 3.6. The Parameter Planes

**Figure 6:**
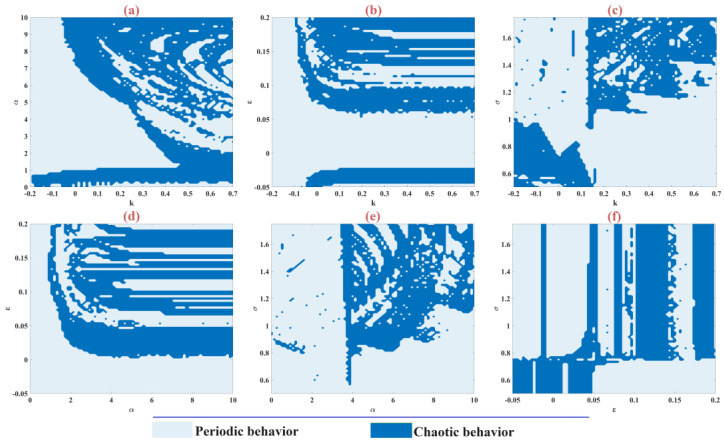
Chaotic and periodic behavior of m-Rulkov model in two-dimensional parameter planes (**a**) for two parameters k and α, (**b**) for two parameters k and ε, (**c**) for two parameters k and σ, (**d**) for two parameters α and ε, (**e**) for two parameters α and σ, (**f**) for two parameters ε and σ, where the value of the parameters is set at α=5, μ=0.1, ε=0.05, σ=1, k=0.46 with random initial conditions.

Figure 6 shows the different behaviors of the m-Rulkov model for the simultaneous changes of parameters $k\in \left[-0.2,\;0.7\right]$ and $\alpha \in \left[0,\;10\right]$ in part a, parameters $k\in \left[-0.2,\;0.7\right]$ and $\varepsilon \in \left[-0.05,\;0.2\right]$ in part b, parameters $k\in \left[-0.2,\;0.7\right]$ and $\sigma \in \left[0.5,\;1.75\right]$ in part c, parameters $\alpha \in \left[0,\;10\right]$ and $\varepsilon \in \left[-0.05,\;0.2\right]$ in part d, parameters $\alpha \in \left[0,\;10\right]$ and $\sigma \in \left[0.5,\;1.75\right]$ in part e, parameters $\varepsilon \in \left[-0.05,\;0.2\right]$ and $\sigma \in \left[0.5,\;1.75\right]$ in part f with random conditions and setting the values of parameter α=5, μ=0.1, ε=0.05, σ=1, k=0.46. So that, the model’s behavior is determined based on the test of zero and one chaos, in which times it is chaotic or periodic. Specifically, the system’s behavior is assessed using a zero-one chaotic algorithm, where a zero indicates periodic behavior and a one signifies chaotic behavior.

## 4. The m-Rulkov Fractional-Order Model

In this section, we use a bifurcation diagram to analyze the discrete fractional-order m-Rulkov map and explore how fractional-order parameters and system parameters influence the model.

### 4.1. Description of Discrete Fractional-Order

We begin by defining discrete fractional order and using it to transform the m-Rulkov model into a discrete fractional-order model. The Caputo-type delta (^C^${∆}_{a}^{q}X\left(t\right)$) represents an order q fractional derivative for $X\left(t\right)$, where $X\left(t\right):N_{a}\to R$ and $N_{a}=\left\{a,\;a+1,\;a+2,\;\ldots \right\}$. It is defined as:

(20) ∆aqCXt=∆a−n−q∆nXt=1Γn−q∑s=at−n−qt−s−1n−q−1∆s−n,

where q∉N denotes the order of the derivative, $t\in N_{a+n-q}$, and $n=\left\lceil q\right\rceil +1$. The fractional sum q for ${∆}_{a}^{-q}X\left(t\right)$ is defined as:

(21) $${∆}_{a}^{-q}X\left(t\right)=\frac{1}{\Gamma \left(q\right)}\sum_{s=a}^{t-q}{\left(t-s-1\right)}^{\left(q-1\right)}X\left(s\right),$$

where $t\in N_{a+n-q}$, q>0, and $t^{\left(q\right)}$ denotes the falling function defined using the gamma function as:

(22) $$t^{\left(q\right)}=\frac{\Gamma \left(t+1\right)}{\Gamma \left(r+1-1\right)}=t\left(t-1\right)\ldots \left(t-q+1\right).$$

For the numerical solution in the discrete map, the fractional order is computed via the fractional difference equation:

∆aqCXt=ft+q−1, xt+q−1,

(23) $${∆}^{k}X\left(t\right)=x_{k},\;n=\left\lceil q\right\rceil +1,\;k=0,\;1,\;2,\;\ldots ,\;n-1,$$

where the equivalent discrete integral is obtained as:

(24) $$x\left(t\right)=x_{0}\left(t\right)+\frac{1}{\Gamma \left(q\right)}\sum_{s=a+n-q}^{t-q}{\left(t-s-1\right)}^{\left(q-1\right)}f\left(s+q-1,x\left(s+q-1\right)\right),\;t\in N_{a+m},$$

where $x_{0}\left(t\right)=\sum_{k=0}^{n-1}\frac{{\left(t-a\right)}^{\left(k\right)}}{\Gamma \left(k+1\right)}{∆}^{k}u\left(a\right)$. According to the definitions for the fractional order, the three-dimensional discrete m-Rulkov model in Equation (5) is formulated as follows:

$$\Delta x(n)=f\left(x(n),y(n)\right)+k\;tanh\left(\varphi (n)\right)\;x(n)-x(n)$$

Δy(n)=−μx(n)

(25) Δφ=εx(n).

After applying the Caputo type delta to it, we obtain the following relationship:

∆avCXtxt=fxt−1−v,yt−1−v+k tanhφt−1−v xt−1−v−xt−1−v,

∆avCyt=−μ xt−1−v,

(26) ∆avCφt=ε xt−1−v.

By expanding Caputo type delta, we have:

$$x\left(t\right)=x\left(a\right)+\frac{1}{\Gamma \left(v\right)}\sum_{s=a+1}^{t-v}{\left(t-s-1\right)}^{v-1}\left[f\left(x\left(t-1-v\right),y\left(t-1-v\right)\right)+k\;tanh\left(\varphi \left(t-1-v\right)\right)\;x\left(t-1-v\right)-x\left(t-1-v\right)\right],$$

$$y\left(t\right)=y\left(a\right)+\frac{1}{\Gamma \left(v\right)}\sum_{s=a+1}^{t-v}{\left(t-s-1\right)}^{v-1}\left[-\mu \;x\left(t-1-v\right)\right],$$

(27) $$\varphi \left(t\right)=\varphi \left(a\right)+\frac{1}{\Gamma \left(v\right)}\sum_{s=a+1}^{t-v}{\left(t-s-1\right)}^{v-1}\left[\varepsilon \;x\left(t-1-v\right)\right],$$

where $\frac{{\left(t-s-1\right)}^{v-1}}{\Gamma \left(\;v\right)}$ is a discrete kernel function and $\frac{{\left(t-s-1\right)}^{v-1}}{\Gamma \left(\;v\right)}=\frac{\Gamma (t-s)}{\Gamma (v)\Gamma (t-s-v+1)}$, a discrete m-Rulkov fractional-order model is constructed for a=0 [43].

$$x\left(n\right)=x\left(0\right)+\frac{1}{\Gamma \left(v\right)}\sum_{j=1}^{n}\frac{\Gamma (n-j+v)}{\Gamma (n-j+1)}\left[f\left(x\left(j-1\right),y\left(j-1\right)\right)+k\;tanh\left(\varphi \left(j-1\right)\right)\;x\left(j-1\right)-x\left(j-1\right)\right],$$

$$y\left(n\right)=y\left(0\right)+\frac{1}{\Gamma \left(v\right)}\sum_{j=1}^{n}\frac{\Gamma (n-j+v)}{\Gamma (n-j+1)}\left[-\mu \;x\left(j-1\right)\right],$$

(28) $$\varphi \left(n\right)=\varphi \left(0\right)+\frac{1}{\Gamma \left(v\right)}\sum_{j=1}^{n}\frac{\Gamma (n-j+v)}{\Gamma (n-j+1)}\left[\varepsilon \;x\left(j-1\right)\right].$$

To expand the range of n in numerical simulations, the following equation is used [44]:

(29) $$\frac{\Gamma \left(n-i+v\right)}{\Gamma \left(n-i+1\right)}=e^{\ln\Gamma \left(n-i+v\right)-\Gamma \left(n-i+1\right)}.$$

The key difference between the integer-order map in Equation (26) and the fractional-order map in Equation (28) lies in the discrete kernel function and the dependence of $x\left(n\right)$ and $y\left(n\right)$ on past values $x\left(0\right),...,\;x\left(n-1\right)$ and $y\left(0\right),\;\ldots ,\;y\left(n-1\right)$. Thus, the current state is influenced by all previous states, demonstrating the memory effects inherent in discrete maps [45].

### 4.2. Dynamic Behaviour

**Figure 7:**
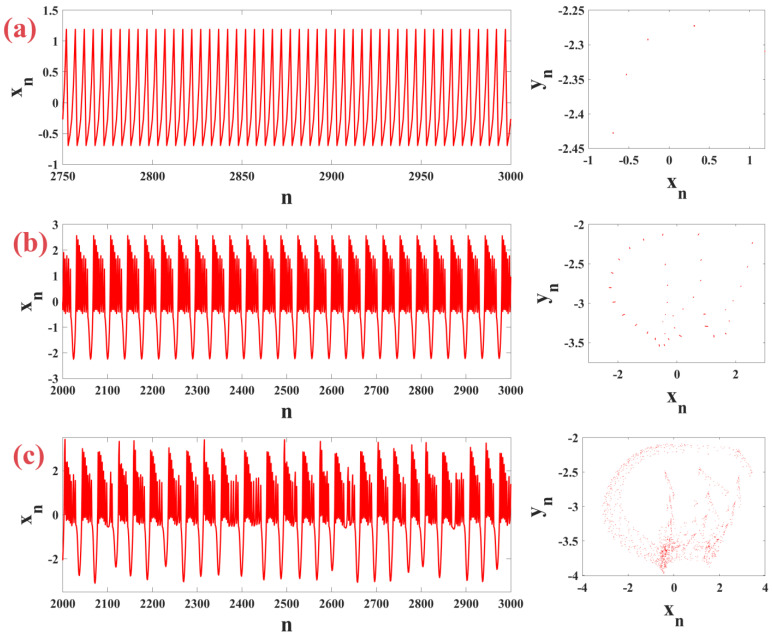
Time series and phase plane representation of neural firing patterns generated by m-Rulkov fractional-order model with constant q=0.875, μ=0.1, ε=0.05, σ=1, k=0.46 in (**a**) tonic firing mode for α=3.41, (**b**) periodic firing mode for α=4.63, and (**c**) chaotic firing mode for α=5.

In this section, we adjust the system parameters to show that the m-Rulkov fractional-order model has the behaviors of tonic firing, periodic firing, and chaotic intermittent firing. By setting q=0.875, μ=0.1, ε=0.05, σ=1, k=0.46 and changing the α parameter, the time series and phase plane of different neural firing patterns are shown by the m-Rulkov fractional-order map in Figure 7. Part (a) shows tonic firing for a neuron with α=3.41 as a continuous and regular firing pattern, part (b) shows periodic firing for a neuron with α=4.63, and part (c) shows chaotic intermittent firing for a neuron with α=5.

### 4.3. Effect of the System Parameter

**Figure 8:**
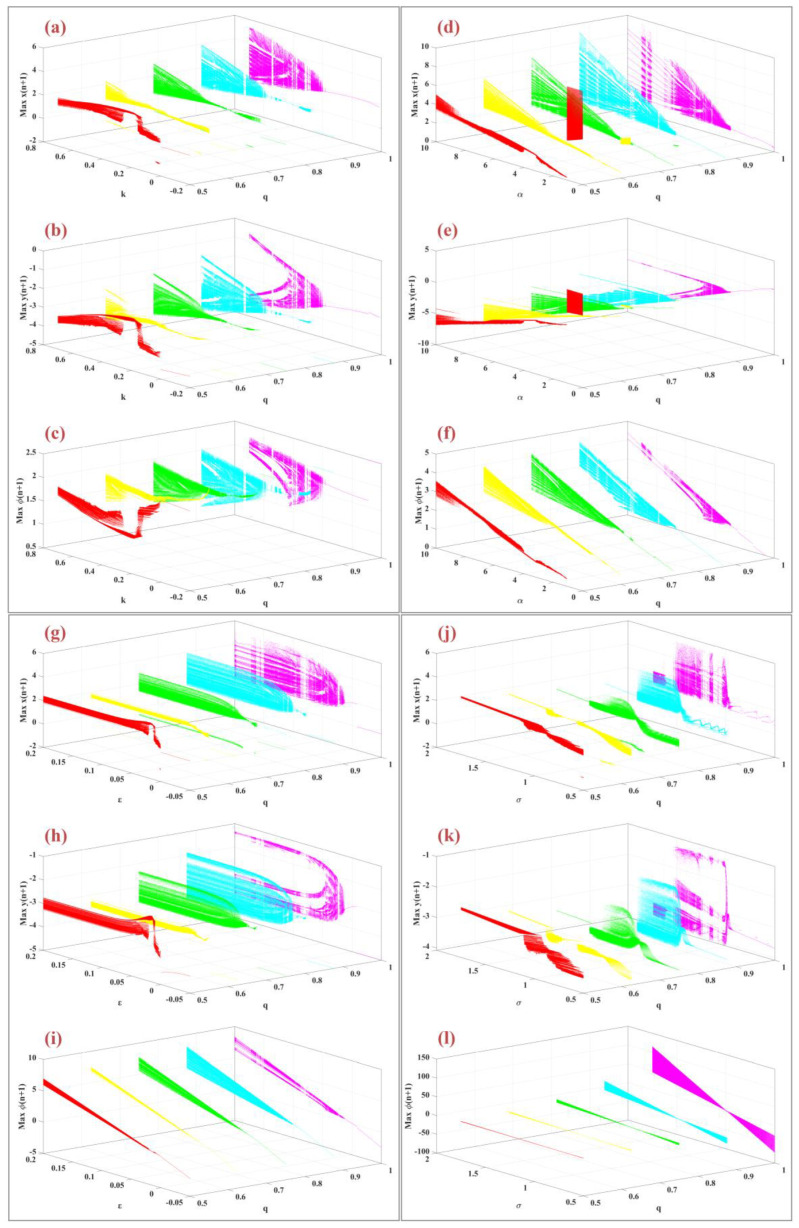
It shows the bifurcation diagram of the variables $x\left(n\right)$, $y\left(n\right)$, $\varphi \left(n\right)$ according to the order of the fraction (q) for the variables (**a**–**c**) *K* = [−0.2, 0.7], (**d**–**f**) *α* = [−0.2, 0.7], (**g**–**i**) *ε* = [−0.2, 0.7], (**j**–**l**) *σ* = [−0.2, 0.7] with random initial conditions.

Operating system parameters play a crucial role in influencing the system’s dynamic behavior. Figure 8 presents the bifurcation diagrams for the variables x(n), y(n), and φ(n) as a function of system parameters and different fractional-order values. In panels (a), (b), and (c), the bifurcation diagrams are plotted for varying values of the parameter K∈[−0.2, 0.7] and fractional orders q=0.5, 0.625, 0.75, 0.875, 1 with other parameters held constant. Panels (d), (e), and (f) depict the bifurcation diagrams for changes in the parameter α within the range [0, 10], with constant parameters μ=0.1, ε=0.05, σ=1, and k=0.46. Panels (g), (h), and (i) illustrate the bifurcation diagrams as the parameter ε varies within the range [−0.05, 0.2]. Finally, panels (j), (k), and (l) show the bifurcation diagrams for changes in the parameter σ within the range [0.5, 2]. In all these diagrams, as the fractional-order q increases, the dynamic behavior becomes more complex, with chaotic states appearing over a wider range of parameters.

### 4.4. Effect of the Fractional Order

**Figure 9:**
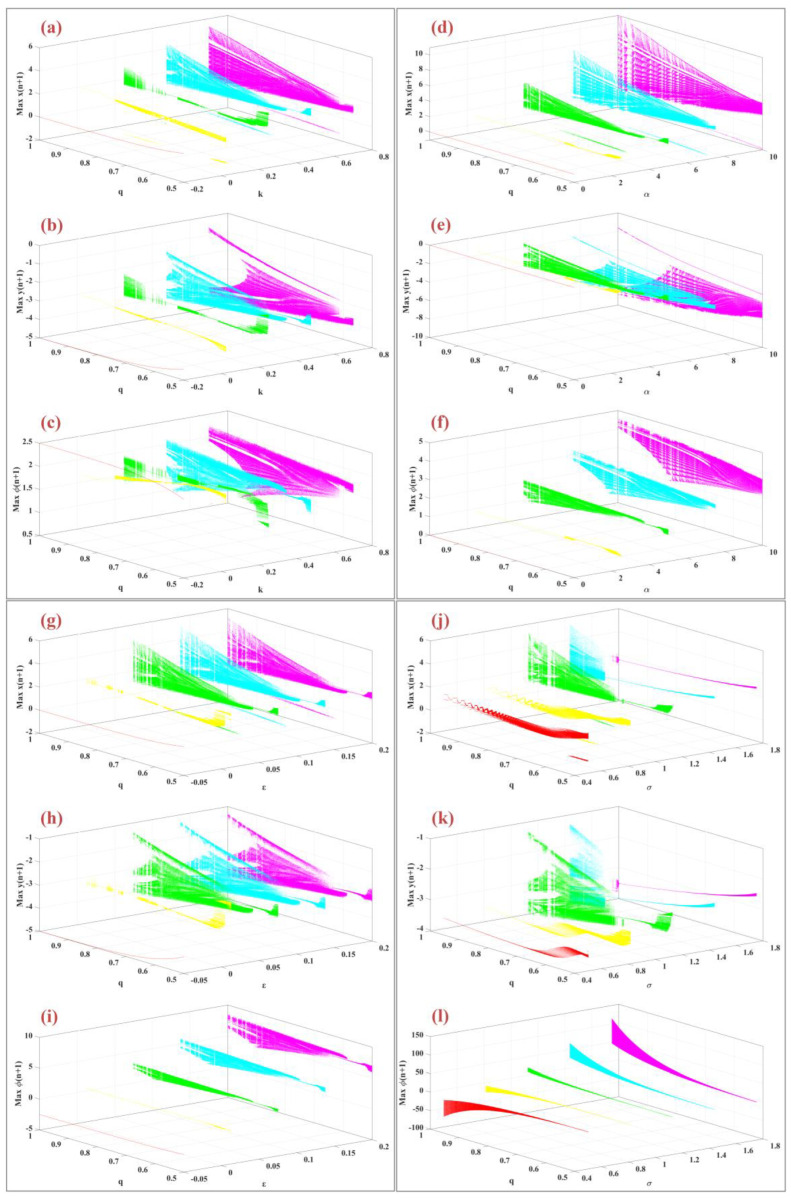
Bifurcations diagram of model parameters for variables x(n), y(n), φ(n) according to the system parameter for different fractional order q∈[0.5, 1], (**a**–**c**) for variable values k=−0.2, 0.025, 0.25, 0.475, 0.7, (**d**–**f**) for values of variable α=0, 2.5, 5, 7.5, 10, (**g**–**i**) for values of variable ε=−0.05, 0.0125, 0.075, 0.1375, 0.2, (**j**–**l**) for values of variable σ=0.5, 0.8125, 1.125, 1.4375, 1.75 are shown.

The fractional-order parameter is a key factor influencing the system’s overall dynamics. Figure 9 illustrates the bifurcation diagrams of the model with respect to fractional order across various parameter values. Panels (a), (b), and (c) display the bifurcation diagrams for the m-Rulkov model as the fractional order q varies within the interval [0.5, 1] for different values of k=−0.2, 0.025, 0.25, 0.475, 0.7. As k increases, the system exhibits enhanced chaotic behavior, a phenomenon referred to as “anti-uniformity” [46]. Panels (d), (e), and (f) show bifurcation diagrams based on fractional order q for various values of α=0, 2.5, 5, 7.5, 10, with parameters fixed at μ=0.1, ε=0.05, σ=1, k=0.46. Panels (g), (h), and (i) present bifurcation diagrams for different values of ε=−0.05, 0.012, 0.075, 0.1375, 0.2, with parameters set at α=0.46, μ=0.1, σ=1, k=0.46. Finally, panels (j), (k), and (l) illustrate the bifurcation diagrams for various values of σ=0.5, 0.8125, 1.125, 1.4375, 1.75, with fixed parameters α=0.46, μ=0.1, ε=0.05, k=0.46.

## 5. Synchronization m-Rulkov Fractional-Order Model

**Figure 10:**
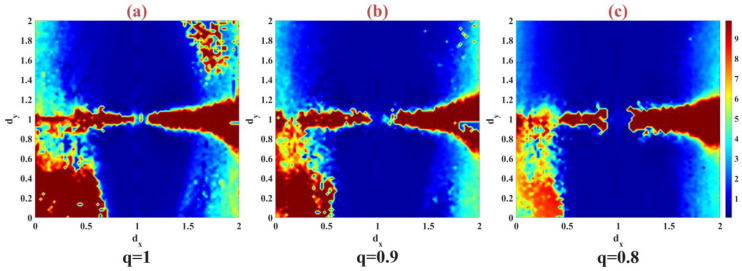
Synchronization error of two discrete fractional-order models of the m-Rulkov neuron model with the change of coupling strength in the interval $d_{x}\in [0,\;2]$ and $d_{y}\in [0,\;2]$ for (**a**) q=1, (**b**) q=0.9, (**c**) q=0.8.

Synchronization is one of the prominent characteristics of the collective in neural networks, during which synchronized temporal activities are observed in neurons. The vital mechanism of synchronization is considered to understand the function of neural networks, transmission, and encoding of information in the brain [29,47,48]. Previously, the effects of electromagnetic induction were observed in a single Rulkov neuron, and in the following, we investigate the existing synchronization between two m-Rulkov neurons in the fractional-order mode. Simulating the effects of electromagnetic induction between two neurons creates a critical challenge due to their simultaneous interaction. This section discusses the simultaneity of these interactions. An electromagnetic current is induced during neuronal interaction and data exchange in a neural network due to the potential difference between two neurons. By this approach, it is possible to simulate the electromagnetic induction current appearing in the synapses by the current passing through the discrete memristor through the electrical coupling power in a two-way coupled topology. For two coupled m-Rulkov neurons, the fractional order is modeled as follows based on Equation (5):

$$x_{1}\left(n\right)=\left[x_{1}\left(0\right)+\frac{1}{\Gamma \left(v\right)}\sum_{j=1}^{n}\frac{\Gamma \left(n-j+v\right)}{\Gamma \left(n-j+1\right)}\left[f\left(x_{1}\left(j-1\right),y_{1}\left(j-1\right)\right)+k\;tanh\left(\varphi_{1}\left(j-1\right)\right)\;x_{1}\left(j-1\right)-x_{1}\left(j-1\right)\right]\right]+d_{x}\left(x_{1}\left(n\right)-x_{2}\left(n\right)\right),$$

$$y_{1}\left(n\right)=\left[y_{1}\left(0\right)+\frac{1}{\Gamma \left(v\right)}\sum_{j=1}^{n}\frac{\Gamma \left(n-j+v\right)}{\Gamma \left(n-j+1\right)}\left[-\mu \;x_{1}\left(j-1\right)\right]\right]+d_{y}\left(x_{1}\left(n\right)-x_{2}\left(n\right)\right),$$

$$\varphi_{1}\left(n\right)=\left[\varphi_{1}\left(0\right)+\frac{1}{\Gamma \left(v\right)}\sum_{j=1}^{n}\frac{\Gamma \left(n-j+v\right)}{\Gamma \left(n-j+1\right)}\left[\varepsilon \;x_{1}\left(j-1\right)\right]\right],$$

$$x_{2}\left(n\right)=\left[x_{2}\left(0\right)+\frac{1}{\Gamma \left(v\right)}\sum_{j=1}^{n}\frac{\Gamma \left(n-j+v\right)}{\Gamma \left(n-j+1\right)}\left[f\left(x_{2}\left(j-1\right),y_{2}\left(j-1\right)\right)+k\;tanh\left(\varphi_{2}\left(j-1\right)\right)\;x_{2}\left(j-1\right)-x_{2}\left(j-1\right)\right]\right]+d_{x}\left(x_{1}\left(n\right)-x_{2}\left(n\right)\right),$$

$$y_{2}\left(n\right)=\left[y_{2}\left(0\right)+\frac{1}{\Gamma \left(v\right)}\sum_{j=1}^{n}\frac{\Gamma \left(n-j+v\right)}{\Gamma \left(n-j+1\right)}\left[-\mu \;x_{2}\left(j-1\right)\right]\right]+d_{y}\left(x_{1}\left(n\right)-x_{2}\left(n\right)\right),$$

(30) $$\varphi_{2}\left(n\right)=\left[\varphi_{2}\left(0\right)+\frac{1}{\Gamma \left(v\right)}\sum_{j=1}^{n}\frac{\Gamma \left(n-j+v\right)}{\Gamma \left(n-j+1\right)}\left[\varepsilon \;x_{2}\left(j-1\right)\right]\right],$$

where $d_{x}$ corresponds to the power of the variable electrical connection x(n) and $d_{y}$ corresponds to the power of the variable electrical connection y(n). Also, subtitle 1 corresponds to the first m-Rulkov neuron model, and subtitle 2 corresponds to the second m-Rulkov neuron model. The parameters of the model are set at α=5, μ=0.1, ε=0.05, σ=1, k=0.46. For both gene models, the initial conditions are randomly chosen between −1 and 1. To determine the synchronization level of two neuronal models, considering two neuronal maps with discrete fractional order, the synchronization error is calculated as a criterion for evaluation as follows:

(31) $$Error=\frac{1}{N}\sum_{n=1}^{N}\sqrt{{\left(x_{1}\left(n\right)-x_{2}\left(n\right)\right)}^{2}+{\left(y_{1}\left(n\right)-y_{2}\left(n\right)\right)}^{2}},$$

where N is the number of time series data samples. In the experiment, three fractional values of q=0.8, 0.9, 1 were used to check the synchronization of the model by changing the coupling strength in the interval $d_{x}\in [0,\;2]$ and $d_{y}\in [0,\;2]$ in Figure 10. According to the evaluation, the results show that reducing the fractional order in the neuronal model increases the synchronization in the model. The islands that existed when q=1 based on anti-phase synchronization disappeared at q=0.8.

## 6. Discussion

The study introduces the fractional memristor-based Rulkov neuron model (m-Rulkov) as a significant advancement in the modeling of neuronal dynamics. By integrating fractional-order derivatives with a memristor into the Rulkov map, this model captures the complex behaviors of neurons with greater than traditional integer-order models. The incorporation of fractional calculus enhances the model’s ability to simulate heritable properties and multiscale dynamic firing activities. Our analysis reveals that the m-Rulkov model can reproduce a range of neuronal firing patterns, including tonic firing, periodic firing, and chaotic firing. This versatility in behavior is attributed to the fractional-order dynamics, which better accommodate the nuanced changes in neuronal activity. The bifurcation diagrams and chaos tests demonstrate that fractional-order derivatives introduce a richer and more varied dynamic landscape compared to integer-order models. Specifically, as the fractional order approaches the ideal value, the system exhibits more chaotic modes, reflecting a more nuanced understanding of neuronal dynamics.

In terms of synchronization, the model’s capability to improve the synchronization of coupled neurons is noteworthy. The fractional-order design significantly enhances synchronization compared to traditional models. This improvement is crucial for understanding collective neuronal behaviors and could have implications for designing more efficient neural networks and neuromorphic systems. The observed effects of the memristor and fractional-order derivatives on synchronization and dynamic firing patterns underscore the potential of this model for more accurate and comprehensive neuronal simulations. These findings align with the broader goal of using advanced modeling techniques to capture the complexities of neural dynamics in both biological and artificial systems. In Appendix A, a table of last researches results along with the proposed model is provided.

### 6.1. Limitations

Despite the promising results, several limitations of this study should be acknowledged. The study explores a broad range of parameters, including memristor flux, fractional order, and coupling strength. However, the full parameter space remains vast, and some parameter combinations may lead to unforeseen behaviors not covered in this study. The use of fractional-order derivatives introduces computational challenges. Simulating these dynamics, especially in higher dimensions or with multiple coupled neurons, can be resource-intensive and may limit practical applications. While the model captures many dynamic behaviors effectively, its correspondence with biological neural networks remains theoretical. Validation against empirical data from biological neurons is necessary to confirm the model’s accuracy and relevance. The memristor in this study is modeled with a simplified representation of flux control. Real-world memristors may exhibit additional complexities that could influence the model’s predictions.

### 6.2. Future Work

Future research should address these limitations and expand on the current findings. Conducting comprehensive studies over a broader range of parameters and including more complex interactions between parameters can provide deeper insights into the model’s behavior. Developing more efficient algorithms for simulating fractional-order systems could alleviate computational challenges and make the model more practical for real-time applications. Comparing the m-Rulkov model’s predictions with experimental data from biological neurons could validate its accuracy and enhance its applicability to neuroscience research. Incorporating more detailed memristor models that account for additional real-world complexities can improve the model’s fidelity and relevance. Exploring the application of the m-Rulkov model in artificial neural networks and neuromorphic systems could demonstrate its practical utility in advanced computing systems.

## 7. Conclusions

The introduction of the fractional memristor-based Rulkov neuron model marks a significant advancement in neuronal modeling. By combining fractional-order calculus with memristor dynamics, this model provides a richer and more accurate representation of neuronal behaviors, including complex firing patterns and improved synchronization of coupled neurons. The study highlights the potential of fractional calculus in capturing nuanced dynamics and enhancing our understanding of neural processes. While the model shows great promise, further research is needed to address its limitations and validate its applications in real-world scenarios. Future work should focus on expanding parameter studies, improving computational efficiency, validating against biological data, and exploring practical applications. The continued development of this model could offer valuable insights into both biological neural networks and artificial neural systems, ultimately contributing to advancements in neuroscience and neuromorphic engineering.

## Data Availability

The datasets generated during and/or analyzed during the current study are available from the corresponding author upon reasonable request.

## Appendix A

**Table A1:** A mathematical comparison of the proposed model with Rulkov memristor models of recent years is provided.

Author’s and Year	Rulkov Model	Memristor Model	Dynamic Behavior Generated
Lu, Y.-M., et al.; 2022; [49]	2D model: $\left\{\begin{array}{c} x\left(n+1\right)=\frac{\alpha }{1+x{\left(n\right)}^{2}}+\beta \\ y\left(n+1\right)=y\left(n\right)+\varepsilon x\left(n\right) \end{array}\right.$	$\left\{\begin{array}{c} i\left(n\right)=\tanh\left(\varphi \left(n\right)\right)v\left(n\right)\\ \varphi \left(n+1\right)=\varphi \left(n\right)+\varepsilon v\left(n\right) \end{array}\right.$	The behavior of the Rulkov neuron in response to electromagnetic radiation is simulated using a newly introduced discrete memristor. They analyze the system’s integer and fractional order properties through bifurcation diagrams, Lyapunov exponents, and recurrence plots. The findings indicate that the fractional order system exhibits more complex dynamics compared to the integer order system, including increased chaos, multiple stable states, and transient chaotic behavior.
Lu, Y., C. Wang, and Q. Deng; 2022; [50]	1D model: $x\left(n+1\right)=\frac{\alpha }{1+x{\left(n\right)}^{2}}+\gamma$	$\left\{\begin{array}{c} i\left(n\right)=\sin\left(\varphi \left(n\right)\right)v\left(n\right)\\ \varphi \left(n+1\right)=\varphi \left(n\right)+\varepsilon v\left(n\right) \end{array}\right.$	They investigate the properties of a two-neuron Rulkov map coupled with memristor elements and a multi-neuron Rulkov neural network. Various numerical simulation techniques, including normalized average synchronization error, bifurcation diagrams, phase portraits, and spatial and temporal patterns, are employed in the analysis. The results reveal that in neural networks coupled with discrete memristors, the system dynamics are influenced by parameters and coupling factors, leading to complex and intriguing behavioral changes.
Bao, H., et al.; 2023; [51]	2D model: $\left\{\begin{array}{c} x\left(n+1\right)=\frac{\alpha }{1+x{\left(n\right)}^{2}}+y\left(n\right)\\ y\left(n+1\right)=y\left(n\right)-\sigma x\left(n\right)-\beta \end{array}\right.$	$\left\{\begin{array}{c} i\left(n\right)=v\left(n\right)\sin\left(\varphi \left(n\right)\right)\\ \varphi \left(n+1\right)=\varphi \left(n\right)+\varepsilon v\left(n\right) \end{array}\right.$	They explore the memristor’s dynamic impact on the neuron model by analyzing bifurcation diagrams and firing patterns. Their findings demonstrate that the proposed Rulkov neuron model effectively captures a range of neuron firing patterns and can generate hyper-chaotic attractors that are significantly influenced by the memristor’s initial value. This suggests that the introduced memristor is highly beneficial for enhancing the original neuron model.
Li, Y., et al.; 2023; [52]	3D model: $\left\{\begin{array}{c} x\left(n+1\right)=\frac{\alpha }{1+x{\left(n\right)}^{2}}+y\left(n\right)+b\tanh\left(z\left(n\right)\right)x\left(n\right)\\ y\left(n+1\right)=y\left(n\right)-cx\left(n\right)\\ z\left(n+1\right)=z\left(n\right)+x\left(n\right) \end{array}\right.$	$\left\{\begin{array}{c} i\left(n\right)=\tanh\left(\varphi \left(n\right)\right)v\left(n\right)\\ \varphi \left(n+1\right)=\varphi \left(n\right)+v\left(n\right) \end{array}\right.$	In their proposed memristor Rulkov neuron, chaotic firing is controlled locally, with the range of chaos adjustable through two independent controllers. These controllers allow direct manipulation of amplitude and frequency. Additionally, the system compensates for complex enhancer-perturbative dynamics, enabling the initial membrane potential to access various self-reproducing attractors and modulate complex firing patterns. This indicates a coexistence of both homogeneous and heterogeneous polystability.
Li, H. and F. Min; 2024; [53]	2D model: $\left\{\begin{array}{c} x\left(n+1\right)=\frac{\alpha }{1+x{\left(n\right)}^{2}}+y\left(n\right)\\ y\left(n+1\right)=y\left(n\right)-\beta x\left(n\right)-\gamma \end{array}\right.$	$\left\{\begin{array}{c} i\left(n\right)=\frac{1}{1+e^{\varphi \left(n\right)}}v\left(n\right)\\ \varphi \left(n+1\right)=\varphi \left(n\right)+\delta v\left(n\right) \end{array}\right.$	They have examined the spatio-temporal patterns, snapshots, and recurrence graphs of nodes in a large-scale discrete Rulkov star-loop neural network model. Their analysis reveals a variety of behaviors in the network, including pseudo-two-well, asynchronous, multi-cluster, single, synchronized, and continuous traveling wave modes. Additionally, they investigate how memory coupling strength and initial conditions affect network behaviors using three metrics: root mean square deviation, average correlation coefficient, and normalized time-averaged synchronization error.
Cao, H., et al.; 2024; [54]	2D model: $\left\{\begin{array}{c} x\left(n+1\right)=\frac{\gamma }{1+x{\left(n\right)}^{2}}+y\left(n\right)\\ y\left(n+1\right)=y\left(n\right)-µ\left(x\left(n\right)-\sigma \right) \end{array}\right.$	$\left\{\begin{array}{c} i\left(n\right)=\left(\alpha \cos\left(\pi \varphi {\left(n\right)}^{2}\right)+\beta \right)v\left(n\right)\\ \varphi \left(n+1\right)=\varphi \left(n\right)+hv\left(n\right) \end{array}\right.$	They employed various methods and parameters to investigate the dynamic behaviors of the discrete memristor map in the context of discrete Chialvo and Rulkov neuron coupling. Their analysis utilized phase diagrams, recurrence plots, bifurcation diagrams, Lyapunov power spectra, and spectral entropy complexity. The study observed various phenomena, including turbulent, chaotic, and periodic attractors, as well as various hidden and simultaneous firing modes. Additionally, they found state transitions and the coexistence of attractors in different types of hyperchaotic regimes.
Ding, D., et al.; 2024; [55]	2D model: $\left\{\begin{array}{c} x\left(n+1\right)=F\left(x\left(n\right),y\left(n\right)\right)\\ y\left(n+1\right)=y\left(n\right)-µ\left(x\left(n\right)-\sigma +1\right) \end{array}\right.$ $F\left(x\left(n\right),y\left(n\right)\right)=\left\{\begin{array}{cc} \begin{array}{c} -\frac{\rho^{2}}{4}-\rho +y\\ \rho x+{\left(x+1\right)}^{2}+y \end{array} & \begin{array}{c} x\left(n\right)\leq -1-\frac{\rho }{2}\\ -1-\frac{\rho }{2}\leq x\left(n\right)\leq 0 \end{array}\\ \begin{array}{c} 1+y\\ -1 \end{array} & \begin{array}{c} 0\leq x\left(n\right)\leq 1+y\\ x\left(n\right)\geq 1+y \end{array} \end{array}\right.$	$\left\{\begin{array}{c} i\left(n\right)=\left(\alpha +\beta \varphi^{2}\left(n\right)\right)v\left(n\right)\\ \varphi \left(n+1\right)=\varphi \left(n\right)+\varepsilon v\left(n\right) \end{array}\right.$	They explore a fractional-order Rulkov neuron model with a discrete memristor subjected to external electromagnetic radiation. The effect of electromagnetic radiation is simulated by fluctuating magnetic flux passing through the neuron membrane. The fractional-order Rulkov neural model dynamics are analyzed using phase attractors, maximum Lyapunov exponents, bifurcation diagrams, and other methods. Additionally, they employ a technique that integrates discrete wavelet transforms, discrete cosine transforms, and chaos index interpolation for their analysis.
Proposed Model	2D model: $\left\{\begin{array}{c} x\left(n+1\right)=f\left(x\left(n\right),y\left(n\right)\right)\\ y\left(n+1\right)=y\left(n\right)-µ\left(x\left(n\right)-\sigma +1\right) \end{array}\right.$ $f(x\left(n\right),y\left(n\right))=\left\{\begin{array}{cc} \frac{\alpha }{1-x\left(n\right)}+y\left(n\right) & x\left(n\right)\leq 0\\ \alpha +y\left(n\right) & 0<x\left(n\right)<\alpha +y\left(n\right)\\ -1 & x\left(n\right)\geq \alpha +y\left(n\right) \end{array}\right.$	$i\left(n\right)=\tanh\left(\varphi \left(n\right)\right)v\left(n\right)$, $\varphi \left(n+1\right)=\varphi \left(n\right)+\varepsilon v\left(n\right)$	We introduce a discrete fractional-order derivative model for the Rulkov memristor neuron model to explore memory effects within this framework. Our findings indicate that the fractional model provides greater accuracy compared to numerical models and effectively simulates explosive patterns and chaotic phenomena. Additionally, our study of the synchronization between two Rulkov neurons with a fractional discrete memristor reveals that coupling strength and fractional-order parameters have a significant impact on neuronal behavior.
